# The role of botulinum neurotoxin BoNT-A in the management of oily skin and acne vulgaris: A comprehensive review

**DOI:** 10.1097/MD.0000000000037208

**Published:** 2024-02-23

**Authors:** Salman Bin Dayel, Ramadan S. Hussein, Huda H. Gafar

**Affiliations:** a Department of Dermatology, College of Medicine, Prince Sattam Bin Abdulaziz University, Al-Kharj, Saudi Arabia; b Clinical Pathology Department, College of Medicine, Prince Sattam Bin Abdulaziz University, Al-Kharj, Saudi Arabia

**Keywords:** acne vulgaris, botulinum neurotoxin, oily skin, treatment

## Abstract

Oily skin and acne vulgaris are prevalent dermatological conditions with a significant impact on both physical and emotional well-being. Despite numerous available treatments, there is a pressing need for effective, long-term solutions. Botulinum Neurotoxin (BoNT-A) has emerged as a potential therapeutic option. However, existing reviews in this area are often limited. This review aims to comprehensively assess the use of BoNT-A in managing oily skin and acne vulgaris while addressing gaps in previous publications. It integrates the latest research, clinical trials, and case studies to provide an up-to-date analysis of BoNT-A mechanisms of action, efficacy, safety, and long-term outcomes. The review systematically analyzes existing evidence, critically evaluates study strengths and limitations, and explores potential synergies with other treatments. It also examines the safety profile of BoNT-A and its potential long-term effects. This review uncovers promising insights into how BoNT-A affects oily skin and acne vulgaris, including its ability to regulate sebum production, reduce inflammation, and potentially shrink pore size. It provides a comprehensive overview of relevant studies and clinical trials, detailing their methodologies, protocols, measures, and results. Collectively, these studies show significant reductions in sebum production, increased patient satisfaction, and smaller pores following BoNT-A treatment. In conclusion, this review addresses knowledge gaps and provides a comprehensive analysis of BoNT-A as a therapeutic option for oily skin and acne vulgaris. By consolidating evidence and highlighting areas for further investigation, it guides clinicians and researchers toward more effective, personalized treatments for individuals with these dermatological challenges.

## 1. Introduction

Oily skin and acne vulgaris, 2 prevalent dermatological conditions, have long been the focus of extensive research and therapeutic interventions due to their substantial impact on individuals’ quality of life. These conditions, characterized by excessive sebum production and the subsequent development of acne lesions, present multifaceted challenges to both patients and healthcare professionals.^[1]^ While a plethora of therapeutic options exist, ranging from topical treatments to systemic medications, there remains an unmet need for treatments that offer both long-term efficacy and safety. In the quest to address these challenges, the field of dermatology has witnessed a relentless pursuit of novel treatment strategies, leading to the exploration of innovative approaches such as Botulinum Neurotoxin BoNT-A.^[2]^

Botulinum neurotoxin type A (BoNT-A) hinders the release of acetylcholine (Ach) in the synaptic cleft by binding to postsynaptic cholinergic receptors. This action results in decreased muscle tone in neuromuscular junctions and interferes with cholinergic sympathetic nerve function in specific glandular tissues. The capacity of BoNT-A to disrupt cholinergic transmission has prompted research into its potential clinical applications for autonomic disorders characterized by excessive glandular secretion, including conditions like hyperhidrosis and sialorrhea.^[1,2]^ A notable skin exocrine gland is the sebaceous gland (SG), responsible for secreting sebum into hair follicles. Clinical observations have long hinted at the involvement of neuronal control in SG regulation.^[3,4]^ Empirical reports on BoNT-A use for controlling excessive sebum production suggest a possible modulatory effect on the neuroendocrine regulation of SGs.^[5]^

Early research in the realm of acne vulgaris predominantly revolved around topical treatments, antibiotics, and isotretinoin. While these approaches have proven effective for many patients, their limitations, including antibiotic resistance and adverse side effects, have underscored the need for alternative therapeutic options. Likewise, the study of oily skin has predominantly concentrated on skincare products and lifestyle modifications, with limited attention to long-term solutions.^[6]^

The need for a comprehensive review that consolidates and critically assesses the current knowledge surrounding BoNT-A utility in oily skin and acne vulgaris is evident. Our study aims to bridge this gap by providing a comprehensive analysis of the existing literature, synthesizing the latest research findings, and offering a systematic evaluation of BoNT-A as a therapeutic option.

The novelty of our study lies in its holistic approach, encompassing both oily skin and acne vulgaris within a single comprehensive review. By amalgamating evidence from diverse sources, we aim to provide a well-rounded understanding of BoNT-A mechanisms, efficacy, safety profile, and potential synergies with existing treatments.

## 2. Methods

The primary objective of this comprehensive review is to critically assess the utilization of botulinum neurotoxin BoNT-A in addressing oily skin and acne vulgaris concerns. Our specific objectives include: Assessing the mechanisms of BoNT-A action relevant to oily skin and acne vulgaris, reviewing the existing clinical trials and case studies on BoNT-A efficacy in managing these dermatological conditions, analyzing the safety profile and potential long-term effects of BoNT-A treatment, and identifying gaps in current research and suggesting areas for future investigation.

**Figure 1.**
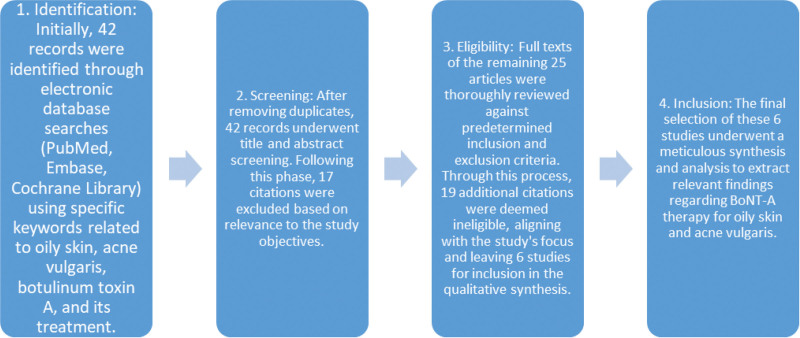
Systematic search and study selection process flowchart (PRISMA flow chart).

Our methodological approach adhered to the PRISMA guidelines (Fig. 1), ensuring a systematic and comprehensive exploration of the available literature. To begin, we formulated a detailed research plan outlining our objectives, criteria for study inclusion/exclusion, search strategy, data extraction methods, and study quality assessment criteria.

Our literature search spanned across electronic databases including PubMed, Embase, and the Cochrane Library, encompassing studies published until June 2023. We included studies regardless of their design and limited the search to English language publications, ensuring a thorough examination of the available evidence. Our search strategy incorporated a combination of keywords pertinent to oily skin, acne vulgaris, botulinum toxin A, and its therapeutic applications.

Two independent reviewers meticulously handled the study selection process, resolving any disparities through consensus. After eliminating duplicates, our initial search yielded 42 records. Following the removal of duplicates, the identified 42 records underwent title and abstract screening. During this phase, 17 citations were excluded based on their lack of relevance to the study’s objectives and focus on BoNT-A therapy for oily skin and acne vulgaris. The remaining 25 articles underwent a thorough assessment of their full texts against predetermined inclusion and exclusion criteria. During this comprehensive review, 19 additional citations were deemed ineligible due to factors such as irrelevant study design, outcome measures not aligned with the research objectives, or lack of focus on BoNT-A therapy for the specified dermatological conditions. Following the meticulous review process, 6 studies emerged as meeting the stringent criteria set for inclusion in the qualitative synthesis and analysis. These studies demonstrated alignment with the study’s focus, providing substantive insights into BoNT-A therapy’s efficacy, mechanisms, safety profile, or long-term effects specifically concerning oily skin and acne vulgaris.

The findings extracted from these selected studies were amalgamated and meticulously organized to address various facets of BoNT-A therapy relevant to the management of oily skin and acne vulgaris. This synthesis culminated in an extensive overview of the available evidence, contributing to a comprehensive understanding of the subject matter.

## 3. Results

### 3.1. Mechanisms of action of BoNT-A relevant to oily skin and acne vulgaris

Oily skin is often characterized by excessive sebum production, contributing to a predisposition to acne. BoNT-A potential in this regard centers on its capacity to regulate sebum production. Recent research has indicated that BoNT-A may modulate sebum production by influencing specific signaling pathways. This modulation may occur by targeting the autonomic nervous system, potentially leading to a reduction in SG activity. Consequently, BoNT-A holds promise in addressing the challenge of excessive sebum production frequently observed in individuals with oily skin.^[7]^

The primary mechanism through which BoNT-A operates is the inhibition of acetylcholine (Ach) release into the synaptic cleft, where it typically binds to muscarinic receptors on the postsynaptic membrane.^[8]^ This inhibition hinders the transmission of signals from nerves to muscles, resulting in muscle relaxation. In the context of SGs, the presence of acetylcholine receptors in sebocytes suggests that BoNT-A action may extend to these cells as well. By blocking acetylcholine activity in sebocytes, BoNT-A may modulate sebum secretion, leading to potential benefits in managing oily skin.^[9]^

ACh operates through 2 primary receptor categories: muscarinic and nicotinic.^[10]^ The muscarinic receptor m2AChR is found in suprabasal sebocytes, and its signaling pathways within the SGs are well-documented.^[11]^ In contrast, the ductal cells of the SGs exhibit the highest levels of alpha 7 nicotinic acetylcholine receptor (α7nAChR) immunoreactivity, which is a major subunit of the nicotinic ACh receptor.^[12]^ Initially identified as a subtype of neuronal receptor, α7nAChR is expressed in various nonneuronal tissues, including the skin. The presence of nonneuronal cholinergic receptors within the SGs suggests that nicotinic signaling pathways and ACh play a role in the pathophysiology of the pilosebaceous unit.^[7,13]^

ACh, in a dose-dependent manner, led to an increase in lipid synthesis through its interaction with α7nAChR, triggering the activation of the ERK signaling pathway, which likely encourages sebocyte differentiation.^[7]^ These findings are in line with immunohistochemical observations that reveal a substantial presence of α7nAChR in mature sebocytes.^[10]^ In vitro experiments with sebocytes obtained from individuals with facial seborrhea showed that these cells are fully mature and exhibit a higher expression of cholinergic receptors. These findings imply that ACh and its receptors have distinct roles in the differentiation of sebocytes and the production of sebum.^[7,14,15]^ It appears that facial seborrhea may be more susceptible to regulation by cholinergic mechanisms compared to normal skin.^[7,16]^ However, it remains to be determined how the human SGs in their natural environment respond to cholinergic stimuli, including whether high doses or prolonged exposure to ACh result in a depolarizing effect and the desensitization of cholinergic receptor neurotransmission.^[16]^

Acne vulgaris, at its core, is an inflammatory condition, with the inflammatory response playing a central role in the development of acne lesions. BoNT-A has displayed anti-inflammatory properties through its ability to inhibit the release of pro-inflammatory neuropeptides. BoNT-A may inhibit the release of substance P, an inflammatory mediator that stimulates lipogenesis in SGs and contributes to inflammation in acne. By reducing substance P activity, BoNT-A may help mitigate inflammation in acne lesions. BoNT-A has demonstrated immunomodulatory properties, including the reduction of pro-inflammatory cytokines. These effects may have a broader impact on the inflammatory cascade in acne. By reducing neurogenic inflammation, BoNT-A may contribute to mitigating acne-related inflammation, potentially limiting the formation of acne lesions and promoting the faster resolution of existing ones.^[16,17]^

Enlarged pores are a common concern among individuals with oily skin, often leading to perceived uneven skin texture. BoNT-A muscle-relaxing effect, which has been well-documented in cosmetic applications, may extend to the pilosebaceous unit. By relaxing the arrector pili muscles surrounding hair follicles within the pilosebaceous unit, BoNT-A could theoretically reduce the tension on these structures, potentially leading to a reduction in pore size.^[17,18]^

## 4. Discussion

### 4.1. Studies and clinical evidence

An overview of these studies offers a comprehensive understanding of their methodologies, treatment protocols, outcome measures, results, and side effects are shown in Table 1.

**Table 1:** Botulinum neurotoxin BoNT-A in the management of oily skin and acne vulgaris.^[2,7,18–21]^

Study	Author	Methodology	Treatment Protocol	Outcome measures	Results	Side effects
Study 1	Shah et al^[18]^	Retrospective analysis of 20 patients. Evaluated safety and efficacy of intradermal injections of BoNT-A for enlarged pore size and sebum excretion.	One Intradermal injection of BoNT-A. 1 month follow-up	Sebum production and pore size after 1 month	About 85% of patients (17/20) were satisfied with oiliness and pore size improvement.	No complications were observed at 1 month.
Study 2	Rose et al^[2]^	A prospective study with 25 patients receiving an intradermal injection of BoNT-A on the forehead with oily skin.	Intradermal injection of BoNT-A. Follow-up at 1 week and 1, 2, and 3 months after injection.	Sebum production, pore size, satisfaction scale at 1 week and 1, 2, and 3 months after injection.	Significant reduction (50%–75% improvement) in sebum production and high patient satisfaction (91%).	Tension decreased in the frontalis muscle in 2 patients.
Study 3	Li et al^[7]^	Split-face-controlled study with BoNT-A on 1 cheek and saline on the other.	BoNT-A injection on 1 cheek, saline on the other of twenty healthy volunteers.	Sebum production, score and pore size at 4 months.	Significant decrease in pore size and sebum score at 4 months in the BoNT-A side compared to the saline control.	Not mentioned.
Study 4	Diamond et al^[19]^	Observation in patients with Tourette syndrome injected with 20–25 units of BoNT-A into paranasal facial expression muscles.	Intramuscular BoNT-A injection.	Facial expression and acne clearance.	Clearance of perinasal acne 1 to 2 weeks posttreatment with improvement persisting for 4 months.	No side effects were mentioned.
Study 5	Min et al^[20]^	Controlled study on BoNT-A treatment for 42 female volunteers with facial wrinkles.	BoNT-A treatment (10 or 20 unit) for facial wrinkles.	Facial wrinkles and sebum production.	Significant sebum reduction at the injection site of both groups. At 16 week follow-up sebum returned to the normal level in both groups.	Mild transient erythema.
Study 6	Matak et al^[21]^	Inhibition of substance P by BoNT-A in animal study (mice).	Injected BoNT/A.	Effect on information.	Potential benefit of BoNT-A in acne due to inhibition of substance P and reduction in inflammation.	Not mentioned.

Collectively, these studies suggest that BoNT-A may offer a promising avenue for the management of oily skin and acne vulgaris. They demonstrate reductions in sebum production, improved patient satisfaction, and decreased pore size following BoNT-A treatment. Although the level of evidence is not consistently mentioned, these findings warrant further investigation to definitively establish the safety and efficacy of BoNT-A in dermatological applications.

### 4.2. Safety profile of BoNT-A

Examining the safety aspects associated with BoNT-A utilization in the context of oily skin and acne vulgaris is paramount, as it informs both clinicians and patients about the risks and benefits of this treatment modality. BoNT-A has a well-established safety record, primarily due to its extensive use in cosmetic and medical applications. However, when considering its use in dermatology for conditions such as oily skin and acne vulgaris, certain safety aspects warrant specific attention.^[22]^

While BoNT-A is generally considered safe when administered by trained professionals, it is not devoid of potential side effects. In dermatological applications, side effects may include localized pain, swelling, bruising, and erythema at the injection site. These side effects are typically transient and resolve within a few days to weeks. Clinicians must educate patients about these potential side effects and ensure they have realistic expectations regarding the treatment’s immediate postinjection phase.^[23]^

There are limited accounts detailing the occurrence of iatrogenic botulism caused by an excessive dose of BoNT/A. This particular type of botulism manifests with identical clinical symptoms as those seen in foodborne or wound botulism. These symptoms include symmetrical cranial nerve issues, followed by a symmetric, weakening paralysis of voluntary muscles. In severe cases, this paralysis can escalate to respiratory failure and ultimately lead to death.^[24]^

Facial muscles do not possess a fascia, and similar glands are situated in the skin. Consequently, when the toxin is injected beneath the skin, it can easily spread without encountering any barriers in neighboring areas. While it becomes less concentrated as it moves away from the injection site, it can still affect nearby muscles or glands, leading to undesired effects such as dry mouth if the salivary gland is involved, drooping of the eyelid if the lid elevator is weakened, or impairment of facial muscle function if the facial nerve endings are affected. However, it’s important to note that these reactions are generally mild, temporary, and, in the worst-case scenarios, can be bothersome for the patient, but they are never life-threatening.^[25]^

Side effects following BoNT-A injections for oily skin and acne vulgaris are rare, but their significance should not be understated. Discussing potential side effects with patients is essential, emphasizing that these effects are typically mild and temporary. Proper injection techniques, patient selection, and adherence to recommended dosages are instrumental in minimizing the risk of adverse events.^[26]^

One potential long-term concern is the development of antibodies to BoNT-A, which can reduce treatment efficacy over time. This phenomenon, known as immunogenicity, has been observed in some cases, particularly in patients receiving frequent or high-dose injections. However, the clinical significance of this issue in dermatological applications remains an area of ongoing research.^[26,27]^ Another consideration is the safety of prolonged BoNT-A use. Long-term effects on the skin, including changes in collagen and elastin, require further investigation to assess their relevance and potential impact.^[25]^

The safety profile and side effects of BoNT-A may differ in different age groups. In younger patients, especially minors and adolescents, there might be heightened sensitivity to treatments due to their developing physiology. Therefore, safety considerations must be tailored accordingly to mitigate any potential risks. Additionally, distinguishing between facial and torso acne holds significance as these areas might respond differently to treatments. Facial acne, often more prevalent among adolescents, may necessitate more cautious approaches due to the sensitivity of facial skin. On the other hand, torso acne, common in various age groups, might require different treatment strategies due to the thicker skin and differences in oil production.^[25,26]^

Considering different types of acne across various age groups is essential too. For instance, adolescents commonly experience hormonal acne due to puberty-related changes. Tailoring treatments to address hormonal imbalances alongside acne management could be critical.^[27]^ In contrast, adults might face acne influenced by factors like stress or hormonal fluctuations, requiring treatments aligned with these triggers. Regarding the geriatric population, addressing how age-related physiological changes impact BoNT-A safety in elderly patients. Evaluating BoNT-A efficacy while considering safety concerns related to aging skin and underlying health conditions.^[28]^

For pregnant or breastfeeding individuals, utmost caution is essential due to the potential transfer of substances to the fetus or through breast milk. Limited data on BoNT-A effects in pregnancy necessitates careful consideration of potential risks to the developing baby. Minors, particularly adolescents, may have unique physiological characteristics and heightened sensitivity to treatments. Considering their developmental stage, any intervention must prioritize safety and account for potential impacts on growth and hormonal balance. Individuals with learning difficulties might require tailored approaches in treatment delivery and communication. Specialized care, clear instructions, and potentially alternative methods of conveying information might be necessary to ensure proper understanding and compliance with treatment protocols.^[29,30]^

Customizing treatment plans for these special populations involves multidisciplinary collaboration, considering not only the dermatological aspects but also the broader health implications and potential interactions with existing conditions or medications. Close monitoring and individualized assessments are fundamental to ensure safety and efficacy while addressing their unique needs and potential vulnerabilities. Ultimately, understanding the nuances of acne across age groups and special populations helps customize treatments, ensuring efficacy while minimizing potential adverse effects, especially in younger individuals whose skin might be more sensitive or reactive. Acne can be self-limiting, especially in the young, and that psychological factors are important in counseling patients.

### 4.3. Potential synergies with other treatment modalities

Combining BoNT-A with other treatment modalities may offer enhanced outcomes for individuals with oily skin and acne vulgaris. BoNT-A can complement existing topical therapies, such as retinoids or antimicrobial agents. Combining treatments may address multiple aspects of acne pathophysiology. Chemical peels, when used in conjunction with BoNT-A, may further improve skin texture and reduce pore size, offering a comprehensive approach to skincare. Some laser and light therapies can target SGs and reduce acne lesions. BoNT-A can be integrated into a holistic treatment plan for synergistic effects. BoNT-A may serve as a maintenance therapy to prolong the effects of other treatments, providing enduring relief from oily skin and acne vulgaris.^[28,29]^

### 4.4. Future directions and research gaps in the field of BoNT-A treatment for oily skin and acne vulgaris

An overview of the research gaps is shown in Table 2.

**Table 2:** This table provides a clear overview of the research gaps and proposed future research directions in the field of BoNT-A treatment for oily skin and acne vulgaris.^[30,31]^

Research focus	Existing gaps	Proposed research directions
Standardized protocols and guidelines	Lack of standardized protocols and guidelines.	Develop treatment guidelines for oily skin and acne vulgaris, including dosing and injection techniques.
		Assess the efficacy and safety of different BoNT-A formulations and injection techniques through comparative studies.
Long-Term Safety and Efficacy	Limited long-term safety and efficacy data	Initiate long-term, prospective studies to assess safety, efficacy, and antiaging effects of BoNT-A (longitudinal studies).
		Investigate histological changes in skin due to long-term BoNT-A use, including effects on collagen, elastin, and sebaceous glands (histological studies).
Patient-Centered Outcomes	Lack of in-depth exploration of patient-centered outcomes	Conduct qualitative research, including interviews and surveys, to explore patient experiences and quality of life improvements (qualitative research).
		Investigate the psychosocial impact of BoNT-A treatment on self-esteem, body image, and emotional well-being (psychosocial impact).
Mechanisms of Action	Need for a deeper understanding of molecular mechanisms	Conduct studies to elucidate how BoNT-A modulates sebum production, reduces inflammation, and impacts skin health at the molecular level (molecular studies).
		Investigate BoNT-A effects on neurotransmitter release in sebocytes and its relevance to oily skin and acne vulgaris (neurotransmitter modulation).
Subgroup Analysis	Limited research on efficacy in specific subgroups	Ensure diverse patient populations in trials to assess variations in treatment responses among different ethnic groups and skin types (diversity in clinical trials).
		Identify patient characteristics predicting better treatment outcomes through subgroup analyses (subgroup analyses).
Combination Therapies	Need for more comprehensive research on combinations	Conduct randomized controlled trials to evaluate the effectiveness and safety of combining BoNT-A with other treatments (clinical trials).
		Identify the best treatment combinations and sequences for maximizing outcomes (optimal combinations).
Immunogenicity and Resistance	Poor understanding of long-term implications	Investigate the prevalence of immunogenicity in patients receiving long-term BoNT-A treatment (Immunogenicity studies).
		Explore mechanisms underlying the development of resistance to BoNT-A and potential mitigation strategies (resistance mechanisms).
Psychological aspects of acne	Need for a better understanding of the Psychological impact of acne.	Investigate the psychological impacts of acne, including its effects on self-esteem, emotional well-being, and body image, to provide a comprehensive view of patient experiences.

## 5. Potential limitations and considerations

While this extensive review delves into BoNT-A for oily skin and acne vulgaris, certain limitations warrant consideration. Its applicability may vary due to individual differences in skin types, genetics, and health conditions. Limited specific studies on BoNT-A dermatological use could impact the depth of evidence synthesis. Potential publication bias favoring positive outcomes might skew interpretations. Varied study quality and design elements could affect the overall assessment’s robustness. Long-term effects and risks from extended BoNT-A use for these conditions may not be fully understood due to limited trial durations. Sparse data in pediatric studies lead to less conclusive findings. Deeper exploration of BoNT-A molecular impact on sebum production and inflammation is needed. Lack of diversity in study populations might hinder broad generalizations across different demographics.

## 6. Conclusion

Botulinum Neurotoxin BoNT-A presents a novel approach to oily skin and acne vulgaris management. The multifaceted actions of BoNT-A offer promising avenues for addressing the complex pathophysiology of these conditions, from sebum regulation to inflammation control and beyond. As research in this field continues to evolve, further insights into these mechanisms will guide the development of more effective and personalized treatment strategies for individuals struggling with oily skin and acne vulgaris.

## Acknowledgments

This study is supported via funding from Prince Sattam bin Abdulaziz University project number (PSAU/2023/R/1444).

## Author contributions

Data curation: Ramadan Hussein, Salman Bin Dayel.

Writing—original draft: Ramadan Hussein.

Writing—review and editing: Ramadan Hussein, Huda Gafar.

Supervision: Salman Bin Dayel.

## References

[1] Arbuckle R Atkinson MJ Clark M. Patient experiences with oily skin: the qualitative development of content for two new patient-reported outcome questionnaires. Health Qual Life Outcomes. 2008;6:80–15. 18925946 10.1186/1477-7525-6-80 PMC2577631

[2] Rose AE Goldberg DJ. Safety and efficacy of intradermal injection of botulinum toxin for the treatment of oily skin. Dermatol Surg. 2013;39:443–8. 23293895 10.1111/dsu.12097

[3] Thomas S Conway J Ebling FJ. Measurement of sebum excretion rate and skin temperature above and below the neurological lesion in paraplegic patients. Br J Dermatol. 1985;112:569–73. 3159411 10.1111/j.1365-2133.1985.tb15265.x

[4] Sudy E Urbina F. Unilateral acne after facial palsy. An Bras Dermatol. 2018;93:441–2. 29924238 10.1590/abd1806-4841.20187437 PMC6001108

[5] Shuo L Ting Y KeLun W. Efficacy and possible mechanisms of botulinum toxin treatment of oily skin. J Cosmet Dermatol. 2019;18:451–7. 30697928 10.1111/jocd.12866

[6] Endly DC Miller RA. Oily skin: a review of treatment options. J Clin Aesthet Dermatol. 2017;10:49–55. PMC5605215 28979664

[7] Li ZJ Park SB Sohn KC. Regulation of lipid production by acetylcholine signaling in human sebaceous glands. J Dermatol Sci. 2013;72:116–22. 23849311 10.1016/j.jdermsci.2013.06.009

[8] Dressler D Adib SF. Botulinum toxin: mechanisms of action. Eur Neurol. 2005;53:3. 10.1159/000083259 15650306

[9] Zouboulis CC Baron JM Böhm M. Frontiers in sebaceous gland biology and pathology. Exp Dermatol. 2008;17:542–51. 18474083 10.1111/j.1600-0625.2008.00725.x

[10] Grando SA Zelickson BD Kist DA. Keratinocyte muscarinic acetylcholine receptors: immunolocalization and partial characterization. J Invest Dermatol. 1995;104:95–100. 7528248 10.1111/1523-1747.ep12613582

[11] Zouboulis CC. The brain of the skin: Sebaceous gland. In: Pappas A, eds. Lipids and Skin Health. New York, NY: Springer. 2015:109–25.

[12] Ramot Y Böhm M Paus R. Translational neuroendocrinology of human skin: concepts and perspectives. Trends Mol Med. 2021;27:60–74. 32981840 10.1016/j.molmed.2020.09.002

[13] Stegemann A Böhm M. Targeting the α7 nicotinic acetylcholine receptor—a novel road towards the future treatment of skin diseases. Exp Dermatol. 2020;29:924–31. 32780438 10.1111/exd.14173

[14] Kurzen H Berger H Jäger C. Phenotypical and molecular profiling of the extraneuronal cholinergic system of the skin. J Invest Dermatol. 2004;123:937–49. 15482483 10.1111/j.0022-202X.2004.23425.x

[15] Kurzen H Schallreuter KU. Novel aspects in cutaneous biology of acetylcholine synthesis and acetylcholine receptors. Exp Dermatol. 2004;13:27–30. 10.1111/j.1600-0625.2004.00258.x 15507109

[16] Valente Duarte De Sousa IC. Novel pharmacological approaches for the treatment of acne vulgaris. Expert Opin Investig Drugs. 2014;23:1389–410. 10.1517/13543784.2014.923401 24890096

[17] Shirshakova M Morozova E Sokolova D. The effectiveness of botulinum toxin type A (BTX-A) in the treatment of facial skin oily seborrhea, enlarged pores, and symptom complex of post-acne. Int J Dermatol. 2021;60:1232–41. 33937981 10.1111/ijd.15574

[18] Shah AR. Use of intradermal botulinum toxin to reduce sebum production and facial pore size. J Drugs Dermatol. 2008;7:847–50. 19112798

[19] Diamond A Jankovic J. Botulinum toxin in dermatology—beyond wrinkles and sweat. J Cosmet Dermatol. 2006;5:169–78. 10.1111/j.1473-2165.2006.00250.x 17173593

[20] Min PX Grassetti L Trisliana Perdanasari A. Sebum production alteration after botulinum toxin type A injections for the treatment of forehead rhytides: a prospective randomized double-blind dose-comparative clinical investigation. Aesthet Surg J. 2015;35:600–10. 25825422 10.1093/asj/sju150

[21] Matak I Tékus V Bölcskei K. Involvement of substance P in the antinociceptive effect of botulinum toxin type A: evidence from knockout mice. Neuroscience. 2017;358:137–45. 28673722 10.1016/j.neuroscience.2017.06.040

[22] Roche N Schnitzler A Genêt FF. Undesirable distant effects following botulinum toxin type A injection. Clin Neuropharmacol. 2008;31:272–80. 18836345 10.1097/WNF.0b013e31815cba8a

[23] Eisele KH Fink K Vey M. Studies on the dissociation of botulinum neurotoxin type A complexes. Toxicon. 2011;57:555–65. 21195107 10.1016/j.toxicon.2010.12.019

[24] Coban A Matur Z Hanagasi HA. Iatrogenic botulism after botulinum toxin type A injections. Clin Neuropharmacol. 2010;33:158–60. 20150804 10.1097/WNF.0b013e3181d479e0

[25] Crowner BE Torres-Russotto D Carter AR. Systemic weakness after therapeutic injections of botulinum toxin A: a case series and review of the literature. Clin Neuropharmacol. 2010;33:243–7. 20852412 10.1097/WNF.0b013e3181f5329e PMC3563356

[26] Hefter H Hartmann C Kahlen U. Prospective analysis of neutralizing antibody titres in secondary non-responders under continuous treatment with a botulinum toxin type A preparation free of complexing proteins–a single cohort 4-year follow-up study. BMJ Open. 2012;2:e000646–97. 10.1136/bmjopen-2011-000646 PMC3449271 22864418

[27] Göschel H Wohlfarth K Frevert J. Botulinum toxin A therapy: neutralizing and non-neutralizing antibodies—therapeutic consequences. Exp Neurol. 1997;147:96–102. 9294406 10.1006/exnr.1997.6580

[28] Park J-Y Lee JS Lee SR. Combined treatment with micro-focused ultrasound with visualization and intradermal incobotulinumtoxin-A for enlarged facial pores: a retrospective study in Asians. Clin Cosmet Investig Dermatol. 2023;16:1249–55. 10.2147/CCID.S402001 PMC10198184 37215534

[29] Rasaii S Sohrabian N Gianfaldoni S. Intralesional triamcinolone alone or in combination with botulinum toxin A is ineffective for the treatment of formed keloid scar: a double-blind controlled pilot study. Dermatol Ther. 2019;32:e12781. 30422367 10.1111/dth.12781

[30] Rho N-K Gil Y-C. Botulinum neurotoxin type A in the treatment of facial seborrhea and acne: evidence and a proposed mechanism. Toxins (Basel). 2021;13:817–29. 34822601 10.3390/toxins13110817 PMC8626011

[31] Phan K Younessi S Dubin D. Emerging off-label esthetic uses of botulinum toxin in dermatology. Dermatol Ther. 2022;35:e15205. 34792262 10.1111/dth.15205

